# Fluctuations of Otoacoustic Emissions and Medial Olivocochlear Reflexes: Tracking One Subject over a Year

**DOI:** 10.3390/audiolres12050051

**Published:** 2022-09-14

**Authors:** Malgorzata Pastucha, W. Wiktor Jedrzejczak

**Affiliations:** 1 Institute of Physiology and Pathology of Hearing, ul. Mochnackiego 10, 02-042 Warsaw, Poland; 2 World Hearing Center, ul. Mokra 17, 05-830 Kajetany, Poland

**Keywords:** efferent auditory system, medial olivocochlear reflex, otoacoustic emissions, TEOAE, repeatability, latency, laterality, longitudinal study

## Abstract

The purpose of the study was to measure the variability of transiently evoked otoacoustic emissions (TEOAEs) and the medial olivocochlear reflex (MOCR) over a long period of time in one person. TEOAEs with and without contralateral acoustic stimulation (CAS) by white noise were measured, from which MOCR strength could be derived as either a dB or % change. In this longitudinal case study, measurements were performed on the right and left ears of a young, normally hearing adult female once a week for 1 year. The results showed that TEOAE level and MOCR strength fluctuated over the year but tended to remain close to a baseline level, with standard deviations of around 0.5 dB and 0.05 dB, respectively. The TEOAE latencies at frequencies from 1 to 4 kHz were relatively stable, with maximum changes ranging from 0.5 ms for the 1 kHz band to 0.08 ms for the 4 kHz band. TEOAE levels and MOCR strengths were strongly and negatively correlated, meaning that the higher the TEOAE level, the lower the MOCR. Additionally, comparison of fluctuations between the ears revealed positive correlation, i.e., the higher the TEOAE level or MOCR in one ear, the higher in the second ear.

## 1. Introduction

Otoacoustic emissions (OAEs) are often used in clinical practice to detect auditory dysfunction [1,2]. In adults, they are generally stable when complications such as noise exposure or ototoxic drugs are absent. However, actual longitudinal data on clinically useful evoked OAEs (e.g., [3]) are lacking. The most studied data sets relate to spontaneous OAEs, but these are not clinically useful [4,5,6]. In the clinic, transiently evoked OAEs (TEOAEs) are one of most commonly used. The short-term repeatability of TEOAEs is around 1–2 dB [7], and changes over a few days or a month are similar [7,8]. For longer periods, there are few data. One study involved following a control group in a conservation program, and here, the TEOAEs declined by 0.7 dB over a year [9]. Another study showed that the time–frequency structure of TEOAEs remained stable over a year [10].

OAEs can be used to estimate the strength of the medial olivocochlear reflex (MOCR), which somehow reflects neural feedback between central and peripheral auditory function (reviewed in [11]). The MOCR is currently not part of clinical practice, but there are some studies that show possible applications: for example, it has been shown that the MOCR is reduced in subjects who have noise-induced tinnitus [12], after noise exposure [13], have juvenile diabetes [14], or are children with auditory processing disorder [15]. The short-term repeatability of the MOCR (up to a few days) has been evaluated, and it seems quite stable if high signal-to-noise ratios (SNRs) are maintained [16,17]. Unfortunately the data on longer term stability are scarce. Immediate reliability of MOCR is around 0.1 dB; change within a few days is similar, while change within the month is 0.5 dB [17,18,19,20]. In case of longer periods, there are works for newborns showing the development of auditory system [21], but there seem to be no data for adults. 

The main rationale for the current study is the limited data for longer periods for OAEs but in particular the MOCR. Moreover, there are not many case studies on OAE stability, and the approach here can add some insight into how they behave, i.e., in the present context, what changes can be expected in an individual subject. 

This study aims to answer the following questions: what are the longitudinal changes of TEOAEs during a period of 1 year; what is the fluctuation in MOCR; which frequency bands are more or less stable; and whether the fluctuations between ears are correlated. TEOAE parameters such as response levels, SNRs, latencies, and strength of MOCR expressed in dB and % were of interest.

## 2. Materials and Methods

### 2.1. Case Description

OAE measurements were performed in one normally hearing, adult female (age 26 years) over one year. The measurements were conducted every week starting from May and ending in the same month, a year later. The measurements were taken between 7:30 a.m. and 4:30 p.m., with most measurements made in the morning (81% before midday). A single measurement session took about 20 min, and in total, there were 48 sessions. 

In the first session, the subject’s hearing status was verified by visual inspection of the ear canal and tympanic membrane of both ears, followed by tympanometry, acoustic reflex threshold (ART) measurement, and pure tone audiometry. All tests were conducted in a sound booth.

Pure tone audiometry was performed with the Madsen Astera (GN Otometrics, Taastrup, Denmark). The subject had pure tone thresholds for air conduction better than 25 dB HL between 0.5 and 8 kHz (Table 1). Middle-ear function was examined using the Titan device (Interacoustics, Middelfart, Denmark). Normal middle-ear function was verified using 226 Hz tympanometry (peak pressure between −100 and +100 daPa and peak-compensated static acoustic admittance of 0.2–1.0 mmhos) and ipsilateral and contralateral ARTs (for clicks and 0.5–4 kHz tones). ARTs were above 80 dB SPL, i.e., well above the levels used for the OAE suppression measurements described below. The subject had no history of otologic disease. 

There are several factors that are known to influence OAEs and MOCR, e.g., infections [22], hormonal changes related to menstrual cycle [4], drugs [23], and noise exposure [24]. Therefore they were controlled as much as possible through the year, as they could possibly affect the investigated parameters. The subject did not have any serious infection during the time of measurements, and she did not take any sick leave. The subject’s menstrual cycle was regular, she did not take any medication, and she did not report exposure to loud noise.

### 2.2. Procedures

TEOAEs were measured using an ILO 292-II system, software version ILOv6 (Otodynamics Ltd, Hatfield, UK). Measurements were made in a sound booth. Before a session, the probes were calibrated using the cavity provided by the manufacturer. 

The standard ILO protocol for measuring contralateral suppression of TEOAEs was used: 65 dB peSPL clicks (linear mode) were delivered to one ear and 60 dB SPL noise to the contralateral ear with 2 s on/off time. The responses with and without CAS (CAS+ and CAS−) were stored in two separate buffers. Clicks were delivered at a rate of 50 per second, giving an acquisition window of 20 ms. To minimize stimulus artifacts, the initial part of the response (0–2.5 ms) was automatically windowed out by the system. Each of the two measurements used 250 averages. Note that the ILO system counts one response as a sequence of responses to four stimuli, and there are two response buffers, so 250 averages means that 2000 clicks were used for each condition (with and without CAS). The minimal accepted SNR was 6 dB. However, the priority of this study was to ensure that SNR was as high as possible, so in each session, eight TEOAE measurements were collected (each of 250 averages) and then averaged. Signal parameters were analyzed for global values and in half-octave bands at 1, 1.4, 2, 2.8, and 4 kHz. Responses were filtered over 750–4500 Hz. (This response represented the global value.) A default artifact rejection level of 6 mPa was used. SNR was calculated by subtracting the noise level (in dB) from the response level (in dB). Measures of TEOAE response level, SNR, latency, and TEOAE suppression (MOCR) were used for analysis. 

The latency of signals was evaluated here as the time from stimulus onset to the maximum of the waveform. This was determined using a Hilbert transform [25]. First, the signals were filtered in a half-octave band around the center frequency of the band concerned (i.e., 1, 1.4, 2, 2.8, and 4 kHz). Then, the envelope of the signal was calculated as the magnitude of the analytic signal provided by the Hilbert transform, and the time index of the envelope maximum was taken to be the latency of the signal. The efferent-induced latency shift was calculated as the difference between latencies of TEOAEs for CAS− and CAS+ conditions. 

MOCR was calculated by three methods. The first was by subtracting the response level with CAS from the level without—the raw MOCR magnitude dB measure (MOCR_MD_). The second method was also based on magnitude but expressed in percent [26] and denoted as MOCR magnitude-percent (MOCR_MP_):

(1) $${\mathrm{MOCR}}_{\mathrm{MP}}=100\times \frac{1}{N}\overset{N}{\underset{n=1}{\sum }}(\left|a_{quiet}\left[n\right]\right|-|a_{noise}\left[n\right]|)/\;\frac{1}{N}\overset{N}{\underset{n=1}{\sum }}\left|a_{quiet}\left[n\right]\right|$$

where *N* is the number of samples (for the present study, it was 512), *a_quiet_* is the amplitude of the TEOAE waveform measured without CAS, and *a_noise_* is the amplitude of the TEOAE waveform measured with CAS. The amplitude is calculated as the absolute value of the Fourier transform, with the result expressed in %. The third method took account of phase effects and was based on the percentage change in the time domain waveforms [27,28]:

(2) $${\mathrm{MOCR}}_{T}=100\times \sqrt{\frac{1}{N}\overset{N}{\underset{n=1}{\sum }}{\left(a_{quiet}\left[n\right]-a_{noise}\left[n\right]\right)}^{2}}\;/\sqrt{\frac{1}{N}\overset{N}{\underset{n=1}{\sum }}{\left(a_{quiet}\left[n\right]\right)}^{2}\;}$$

The variables here are the same as for Equation (1), but the amplitude is taken directly from the time waveform, and the result is expressed in %. To sum up, MOCR_MD_ and MOCR_MP_ are based on amplitude change, while MOCR_T_ incorporates amplitude and phase changes of the signal.

To prevent the subject from falling asleep during the tests, which could cause artifacts (e.g., snoring, change of position), a movie was shown with the sound track muted. As shown by recent studies, such an experimental design does not seem to affect the major TEOAE parameters or suppression levels of TEOAEs by CAS [29,30].

The subject had spontaneous OAEs (SOAEs) in both ears, as verified by measurement of so-called synchronized SOAEs (SSOAEs). SSOAEs were acquired using the in-built routine provided by the ILO 292 equipment. OAEs were evoked by click stimuli of 80 dB peSPL and recorded in an 80 ms window, with the first 20 ms of each averaged response (containing largely the evoked part) discarded. The spectra of responses from the last 60 ms were analyzed in search for SSOAEs. An SSOAE was identified when a peak was found in the spectrum that exceeded the noise floor by 6 dB. The subject had four SSOAEs in each ear in the frequency range of 1–2.5 kHz. The highest level of SSOAEs was around –7.7 dB SPL. This information is provided only as a part of description of the subject, as SSOAEs are known to affect OAEs and MOCRs [17], and the presence of SSOAEs was not further analyzed. 

### 2.3. Data Analysis

All analyses were made in Matlab (version 2018b, MathWorks, Natick, MA, USA). For latency change in MOCR, the statistical significance of mean differences was evaluated using repeated measures analysis of variance (rmANOVA). Post hoc tests were conducted using a *t*-test (when the data fulfilled a criterion of normality); otherwise, a Wilcoxon rank sum test was used. For some analyses, Pearson correlations also were calculated. As a criterion of significance, a 95% confidence level (*p* < 0.05) was chosen. When conducting multiple comparisons, *p*-values were adjusted using the Benjamini–Hochberg [31] procedure to control for false-discovery rates.

## 3. Results

**Figure 1:**
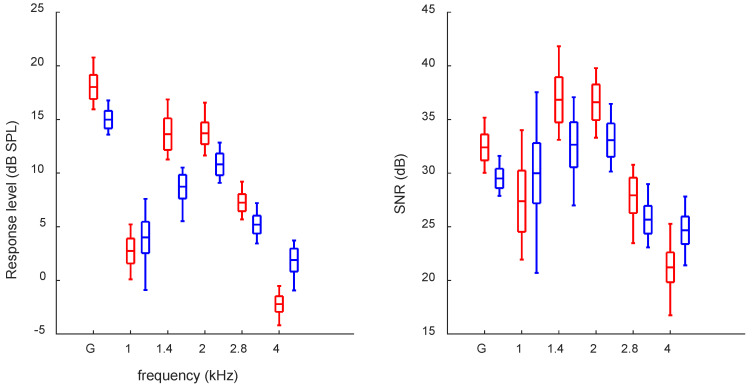
Average response levels (left panel) and SNRs (right panel) for TEOAEs without CAS measured over one year for left and right ears (blue and red, respectively). Boxes depict the mean and standard deviations, and the whiskers show the minimum and maximum. G, global (broadband).

The average TEOAE response levels and SNRs (without CAS) are shown in Figure 1. The global (broadband) response level fluctuated over the year between 15.9 and 20.8 dB for the right ear and between 13.6 and 16.8 dB for left. The fluctuations for half-octave frequency bands were higher, especially for 1 and 1.4 kHz. SNR fluctuated between 30.0 and 35.1 dB for the right ear and between 27.9 and 31.6 dB for the left. 

**Figure 2:**
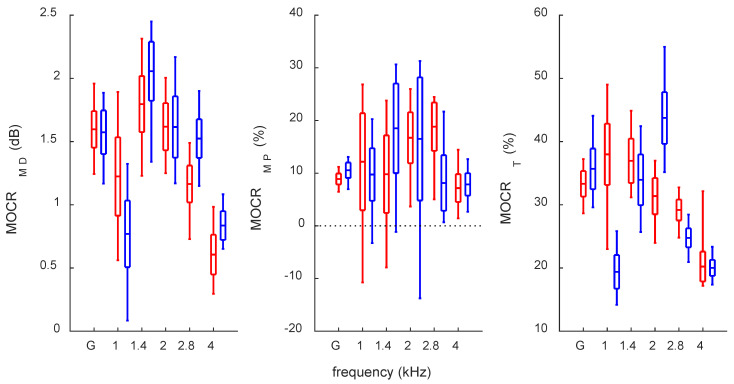
Average MOCR for TEOAEs. All other descriptions as in Figure 1.

For MOCR, the fluctuations over one year for the three measures are shown in Figure 2. For all measures, the global values exhibited the lowest spread, while there were quite high fluctuations for half-octave frequency bands of 1, 1.4, and 2 kHz. Of particular interest, note that for the MOCR_MP_, in these three bands (center plot in Figure 2), there are multiple occasions when the MOCR is negative; i.e., contralateral noise causes OAE enhancement rather than inhibition. 

The global MOCR_MD_ fluctuated over one year from 1.24 to 1.96 dB for the right ear and from 1.17 to 1.89 dB for the left. The global MOCR_MP_ fluctuated over one year from 6.47 to 11.2% for right ear and from 6.96 to 13.08% for left ear. The global MOCR_T_ fluctuated over one year from 28.6 to 37.2% for the right ear and from 29.6 to 44.1% for the left.

**Figure 3:**
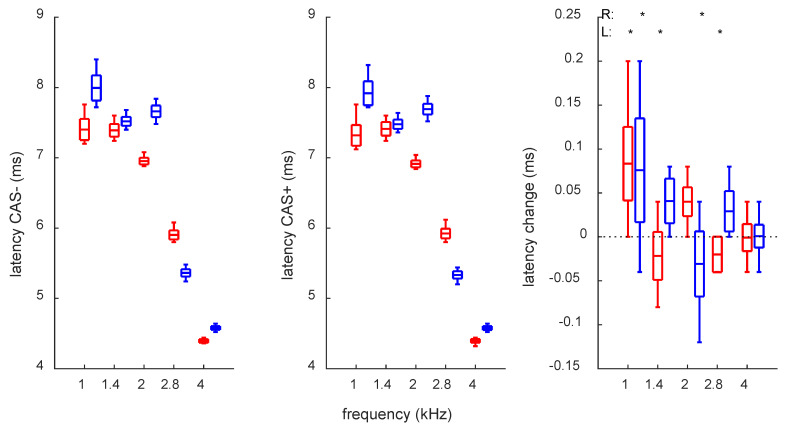
Average latency for TEOAEs and MOCR induced latency change. * Asterisks in the right plot mark significant differences from zero. All other descriptions as in Figure 1.

Figure 3 presents fluctuations over one year in latency across different frequency bands. It should be underlined that since the signal was 512 samples long, and sampling was at 25 kHz, the difference between time points was 0.04 ms, so there is no way of detecting changes smaller than 0.04 ms with this system. As expected from previous studies, the latency follows a pattern of longer values for lower frequencies and shorter values for higher frequencies [32]. The fluctuations of the latency were small, spanning from around 0.5 ms for the 1 kHz band to 0.08 ms for the 4 kHz band.

An rmANOVA was used to examine differences between latencies of TEOAEs as a function of frequency and of test (CAS+ and CAS− conditions). It was found that there was a significant effect of frequency (for right ear: *F*(4, 235) = 10,275, *p* < 0.001; for left ear: *F*(4, 235) = 13,152, *p* < 0.001). Similarly, for test, the values were: for right ear, *F*(1, 235) = 92, *p* < 0.001; and for left ear, *F*(1, 235) = 104, *p* < 0.001. There was also an interaction of frequency and test: for right ear, *F*(4, 235) = 142, *p* < 0.001; for left ear, *F*(4, 235) = 63, *p* < 0.001.

Interestingly, there was no consistent shift in both sorts of latencies (right panel of Figure 3). For some frequencies, the shift was toward shorter latencies after CAS application (1 kHz), while for other frequencies, the shift was to longer latencies. Moreover, the shift was not consistent between the ears so that sometimes, in one ear, the latency became shorter with CAS, while in the other, CAS caused latency to increase. 

TEOAE waveforms usually have a few “ripples” (maxima of envelope) for each frequency band (see, e.g., Figure 2 in [25]). Therefore, latencies were derived from the ripple of highest magnitude. However, in some instances (about 10%, mostly for 1 kHz band), the highest magnitude ripple was different (e.g., most often, the first one but sometimes the second one). This switching between ripples is explained in Figure 2 in ref. [25] in case of responses to different levels of stimulation. However, here, we observed switching for the stimuli of the same magnitude measured in different weeks throughout a year. For analyses, only the latency of the same (most frequent) ripple was taken.

Table 2 summarizes changes from week to week and month to month between response levels, SNRs, latencies, and MOCRs. Additionally, a final comparison between first and last month (after 1 year) is shown. Latency in the 1 kHz band is specified, as the MOCR-induced shift was biggest in this band. It can be seen that while weekly/monthly changes of response level may be up to around 3 dB, the changes after one year are very small, as low as 0.1 dB. A similar pattern occurs with MOCR_MD_ and MOCR_MP_, with minimum changes of around 0.2 dB or 1%. Slightly differently behavior was observed for MOCR_T_, which shows greatest changes over time, with a minimum of around 1% but with the maximum reaching even 9%. This show that while the MOCR effect is greatest by MOCR_T_ estimate, the fluctuations of MOCR_T_ are also higher than MOCR_MD_ and MOCR_MP_.

Table 3 shows correlations between right and left ear for response levels, SNRs, and MOCRs throughout the whole year. The response levels correlated strongly between ears, while MOCR_MD_ correlated weakly, MOCR_MP_ correlated moderately, and the MOCR_T_ correlation was not significant. The latency fluctuations were also correlated with response levels (for right ears: *r* = 0.84, *p* < 0.001, for left ears: *r* = 0.7, *p* < 0.001).

Table 4 shows correlations between response levels and different MOCR estimates throughout the whole year. The response levels correlated negatively with MOCR. The correlations were not significant for one ear for MOCR_MD_ and MOCR_T_ estimates. The most consistent results were for MOCR_MP_, for which the MOCR strongly and negatively correlated with response level; i.e., the higher the response level, the lower the MOCR.

Table 5 shows correlations between TEOAE latency at 1 kHz and different MOCR estimates throughout the whole year. The latency correlated negatively with all MOCR estimates except MOCR_T_ for the left ear. 

We found no periodic fluctuations in TEOAE or MOCR over the year related to menstrual cycle, season, or time of day (although measurements were done only between 7:30 a.m. and 4:30 p.m.).

## 4. Discussion

This study has shown that TEOAE magnitude and MOCR strength fluctuate over a year but, like a chaotic system, tend to orbit a stable attractor. The cause of fluctuations remains unknown. We can hypothesize that they reflect day-to-day changes in functioning, similar to how other organs fluctuate depending on their environment. It is possible that OAEs are somehow related to whole body function; for example, there are reports showing the relationship between OAEs and body temperature, CSF pressure, and heart rate [4,33,34].

The highest response levels and SNRs for this subject were in the range of 1.4–2 kHz. Similarly, the strongest MOCR were in the same range, and this is generally consistent with what is observed for other subjects [17]. However, the greatest variability was in that same range, together with the addition of the 1 kHz half-octave frequency band. This differs from a larger group study, which showed smaller variability in the 1–2 kHz bands compared to the 2.8 and 4 kHz bands. 

It should be underlined that this individual had “strong” OAEs with a high SNR ratio, which is typical for young female subjects with normal hearing [35]. She also exhibited SSOAEs in both ears, and subjects with SSOAEs also exhibit TEOAEs with high response levels, SNRs, and MOCRs [17,23]. This is not generally the case for all normally hearing subjects. A considerable fraction of subjects with audiometrically normal hearing have OAEs that do not pass typical screening criteria (e.g., 6 dB SNR). This applies particularly to male subjects whose OAEs are likely to be weaker than for females even though the MOCRs are similar [36]. Furthermore, TEOAE response levels and MOCRs tend to decrease with increasing age [37].

There was also a clear laterality effect in the TEOAEs, with larger global response levels and SNRs for the right ear. This is consistent with the literature [35]. The fluctuations of response levels and SNRs were also slightly higher in the subject’s right ear. This might be related to the stronger response levels in the right ear but might also be a personal feature of the subject. For MOCR, the laterality effects were less clear. There seemed to be no difference between ears for the global MOCR_MD_ estimate, but for MOCR_MP_ and MOCR_T_, there was a lower effect for the right ear (contrary to response levels and SNRs). A similar result has been obtained for group data [36], where fluctuations in MOCR were similar between ears for MOCR_MD_ but lower in right ears for MOCR_MP_ and MOCR_T_.

The study shows that the MOCR_T_ estimate seems to exhibit higher variability than MOCR_MD_ and MOCR_MP_. It was the only MOCR estimate that did not show correlations between the ears. This points to the conclusion that MOCR_T_ seems to be the least reliable estimate of MOCR as measured with TEOAEs. 

In previous studies, the latency changes in MOCRs have been shown to be in the same direction irrespective of frequency (e.g., [38]). Here, however, MOCR latency showed different behavior across frequencies. For the 1 kHz band, both ears showed a decrease in latency, but other frequencies behaved differently, i.e., an increase for some frequencies while a decrease for others (Figure 3, right). The latency fluctuations correlated with fluctuations in TEOAE response level and MOCR, so perhaps these factors play a part in the variability.

A limitation of this study is that it involved only one person, so generalization of the results should be made with caution. Another limitation is that the hearing thresholds and middle-ear status were evaluated only a few times during the year. As the OAEs were generally very stable, and the subject did not report any problems with hearing, there seemed to be no reason to perform additional tests. Prolonging the testing time would have made it more difficult to collect OAE data in a regular way, so shortening the procedure seemed a reasonable tradeoff.

In summary, the present observations point to some interesting properties of longitudinal changes of TEOAEs and MOCR, but further investigations on more subjects are called for. In particular, there seems to be a need to establish standardized norms for OAE-based measures.

## 5. Conclusions

The present study has shown that while the short-term fluctuations of OAE levels and MOCR were quite high, they were generally stable over the long term. After one year, the changes in TEOAE levels were within 0.1–1 dB, and changes in MOCR were within 0.1–0.3 dB. The TEOAE latency was also quite stable, with fluctuations ranging from 0.5 ms for the 1 kHz band to 0.08 ms for the 4 kHz band. Interestingly, the fluctuations of OAE levels, latencies, and MOCRs were correlated between the ears. 

## Figures and Tables

**Table 1:** Pure tone hearing thresholds for studied subject. R, right; L, left.

Ear	Frequency (kHz)										
	0.125	0.25	0.5	0.75	1	1.5	2	3	4	6	8
R	0	−5	−5	0	−5	5	0	−10	−5	−5	0
L	0	−5	−5	−5	−5	−5	5	0	−10	0	5

**Table 2:** Average week-to-week and month-to-month differences (absolute values, unsigned) for global TEOAE response level (dB SPL), SNR (dB), latency (ms at 1 kHz), and global MOCR within consecutive weeks, months, and between first and last month. R, right; L, left; STD, standard deviation.

		Week			Month			First/Last Month		
		Mean (STD)	Min	Max	Mean (STD)	Min	Max	Mean (STD)	Min	Max
Resp. level	R	0.95 (0.73)	0.05	2.67	1.10 (0.85)	0.04	3.33	0.57 (0.45)	0.06	1.15
	L	0.66 (0.52)	0.02	2.31	0.87 (0.75)	0.02	3.04	0.48 (0.35)	0.12	0.94
SNR	R	1.33 (1.01)	0.04	4.18	1.43 (1.25)	0.00	5.05	1.16 (0.65)	0.55	2.08
	L	1.03 (0.75)	0.01	2.67	0.96 (0.74)	0.05	2.53	1.35 (0.69)	0.61	2.00
Latency	R	0.13 (0.11)	0.00	0.52	0.16 (0.12)	0.00	0.48	0.17 (0.11)	0.08	0.32
	L	0.12 (0.12)	0.00	0.36	0.13 (0.12)	0.00	0.48	0.27 (0.11)	0.16	0.40
MOCR_MD_	R	0.15 (0.11)	0.0002	0.52	0.15 (0.11)	0.005	0.40	0.27 (0.05)	0.23	0.34
	L	0.14 (0.11)	0.002	0.40	0.16 (0.11)	0.01	0.42	0.14 (0.07)	0.08	0.24
MOCR_MP_	R	1.00 (0.85)	0.02	3.71	1.17 (0.83)	0.08	3.37	1.63 (0.55)	0.98	2.17
	L	1.06 (0.82)	0.07	3.71	1.31 (1.03)	0.01	3.63	1.14 (0.22)	0.86	1.38
MOCR_T_	R	2.06 (1.25)	0.31	5.62	2.01 (1.63)	0.07	7.18	2.11 (1.58)	1.13	4.46
	L	2.96 (2.12)	0.11	9.02	3.32 (2.31)	0.04	8.12	4.06 (3.93)	1.08	9.36

**Table 3:** Correlations between ears of TEOAE parameters and MOCR estimates (measured over the whole year). Latency measurement derives from 1 kHz band; all other parameters are derived as global (broadband) values. R, right; L, left.

	*r*	*p*
Response level R vs. L	0.72	<0.001
SNR R vs. L	0.30	0.04
Latency R vs. L	0.53	<0.001
MOCR_MD_ R vs. L	0.35	0.01
MOCR_MP_ R vs. L	0.56	<0.001
MOCR_T_ R vs. L	−0.07	0.62
MOCR-induced latency change R vs. L	0.06	0.68

**Table 4:** Correlations between response level and MOCR estimates. R, right; L, left.

		*r*	*p*
Resp. level vs. MOCR_MD_	R	−0.21	0.16
	L	−0.45	<0.001
Resp. level vs. MOCR_MP_	R	−0.66	<0.001
	L	−0.72	<0.001
Resp. level vs. MOCR_T_	R	−0.43	<0.001
	L	0.02	0.90

**Table 5:** Correlations between and TEOAE latency (at 1 kHz) and MOCR estimates. R, right; L, left.

		*r*	*p*
Latency vs. MOCR_MD_	R	−0.41	<0.001
	L	−0.46	<0.001
Latency vs. MOCR_MP_	R	−0.74	<0.001
	L	−0.62	<0.001
Latency vs. MOCR_T_	R	−0.45	<0.001
	L	0.22	0.14

## Data Availability

The data underlying this article will be shared on reasonable request to the corresponding author.
